# “Hurdles on the path to 90-90-90 and beyond”: Qualitative analysis of barriers to engagement in HIV care among individuals in rural East Africa in the context of test-and-treat

**DOI:** 10.1371/journal.pone.0202990

**Published:** 2018-08-30

**Authors:** James Ayieko, Lillian Brown, Sibyl Anthierens, Annelies Van Rie, Monica Getahun, Edwin D. Charlebois, Maya L. Petersen, Tamara D. Clark, Moses R. Kamya, Craig R. Cohen, Elizabeth A. Bukusi, Diane V. Havlir, Carol S. Camlin

**Affiliations:** 1 Center for Microbiology Research, Kenya Medical Research Institute, Nairobi, Kenya; 2 Division of HIV, Infectious Diseases & Global Medicine, University of California San Francisco, San Francisco, California, United States of America; 3 Faculty of Medicine and Health Sciences, University of Antwerp, Antwerp, Belgium; 4 Department of Obstetrics, Gynecology and Reproductive Sciences, University of California San Francisco, San Francisco, California, United States of America; 5 Department of Biostatistics and Epidemiology, University of California Berkeley, Berkeley, California, United States of America; 6 Department of Medicine, College of Health Sciences, Makerere University, Kampala, Uganda; Pontificia Universidade Catolica do Rio Grande do Sul, BRAZIL

## Abstract

**Background:**

Despite substantial progress, gaps in the HIV care cascade remain large: globally, while about 36.7 million people were living with HIV in 2015, 11.9 million of these individuals did not know their HIV status, 12.7 million were in need of antiretroviral therapy (ART) and 13.0 million were not virally suppressed. We sought to deepen understanding of the barriers to care engagement at three critical steps of the care cascade proposed to make greatest impact for attaining the UNAIDS 90-90-90 targets aimed at shutting down the HIV epidemic.

**Methods:**

Analyses were conducted among HIV-infected adults in rural East Africa. Qualitative data were collected using in-depth interviews among 63 individuals participating in an ongoing test-and treat trial (NCT01864683) in its baseline year (July 2013-June 2014). Audio recordings were transcribed, translated into English, and coded using Atlas.ti software. Data were analyzed using a thematic framework for explaining barriers to care engagement that drew upon both theory and prior empirical research in similar settings.

**Results:**

Multiple barriers to engagement in care were observed. HIV-related stigma across dimensions of anticipated, internalized and enacted stigma manifested in denial and fears of disclosure, and influenced lapses in care engagement across multiple steps in the cascade. Poverty (lack of food and transport), lack of social support, work interference, prior negative experiences with health services, drug side effects, and treatment fatigue also negatively affected ART adherence and viral suppression. Gender differences were observed, with work interference and denial disproportionately affecting men compared to women.

**Conclusion:**

Multiple barriers to HIV care engagement still pervade rural sub-Saharan settings threatening the realization of the UNAIDS 90-90-90 targets. To control the epidemic, efforts need to be accelerated to combat stigma. Patient economic empowerment, innovative drug formulations, as well as more patient-responsive health systems, may help overcome barriers to engagement in care.

## Introduction

Antiretroviral therapy (ART) has become more potent, better tolerated, and less complex, enabling people living with HIV (PLWH) adhering well to treatment to achieve viral suppression [1,2]. ART not only improves quality of life [3,4] but also prevents transmission of infection [5,6,7]. Several large trials are underway to test whether the HIV “test-and-treat” strategy [8] can reduce HIV incidence, as suggested by mathematical models [9] and observational studies [10,11]. The 90-90-90 UNAIDS targets envision that if 90% of PLWH are tested, 90% of those tested are initiated on ART, and 90% of those on ART achieve viral suppression, a high population-level viral suppression will be attained [12]—the key to the promise of the test-and-treat strategy to ‘shut down’ the HIV epidemic. For test-and-treat to succeed, early diagnosis coupled with linkage to and retention in ART care is required. Yet in many settings, late and low rates of HIV testing, poor linkage and retention, and suboptimal adherence to ART [13,14,15] continue to impede optimal outcomes. The barriers to effective engagement across every step of the HIV care cascade must be well understood in order to be successfully addressed; this is of greatest importance now as the scale up of test-and-treat takes place in sub Saharan Africa and other developing countries with an aim of achieving the UNAIDS 90-90-90 targets.

In this qualitative study, we sought to better understand barriers to engagement in HIV care, with a particular focus on the testing, ART initiation and viral suppression steps of the cascade, during the baseline year of an ongoing HIV test-and-treat trial, the Sustainable East African Research for Community Health (SEARCH) study (NCT01864683)[16]. These data were collected prior to full implementation of SEARCH, and thus reflect conditions in communities prior to full ‘rollout’ of the test-and treat intervention.

## Methods

### Study design and sampling

Data are from a qualitative study embedded within the SEARCH trial, a community-level cluster randomized HIV test-and-treat trial currently being implemented in 32 rural communities in Uganda (10 in southwestern Uganda, 10 in eastern Uganda) and western Kenya (12). SEARCH aims to increase HIV testing and care uptake through community-led, multi-disease and patient-centered approaches, including community health campaigns followed by tracking and home-based testing, along with streamlined HIV care delivery; study details are published elsewhere [16]. The qualitative study is conducted within 8 of the 32 SEARCH communities: 2 matched intervention and control communities in southwestern Uganda, 2 in eastern Uganda, and 4 in western Kenya. The overall aims of the qualitative study in SEARCH are to ascertain how a large test and treat effort influences community norms, beliefs, attitudes and behaviors related to HIV and, in turn, how these changes influence the uptake and success of this effort. Purposive and stratified random sampling techniques were used to establish three longitudinal in-depth interview cohorts: community members, community leaders, and HIV care providers; this analysis uses only the data collected from HIV-positive members of the community cohort, which was composed via random selection from household rosters established by the SEARCH socio-economic survey. Within strata defined by gender and HIV care status (ascertained during baseline testing in SEARCH), 5 HIV-negative and 9 HIV-positive adults (aged 15 or over) per community (3 with CD4 count above 500 cells/mm^3^ and not on ART, 3 on ART, and 3 eligible for ART by CD4 (<500 cells/mm^3^) but not linked to care at sampling) were selected for recruitment for the qualitative study. A total of 63 HIV-positive individuals were successfully found and consented to enrollment in the study. Data were collected after baseline testing, during the first year of SEARCH (July 2013-June 2014). At the time of interview, among the 63 participants, 42 were engaged in HIV care, with 21 already on ART; a total of 21 were not engaged in care. The median age of the 63 participants was 37 years (IQR 30–44); 34 were female, 46 were married, 36 were from Kenya and 27 were from Uganda.

### Data collection and analysis

In-depth semi-structured interview guides were used for data collection. A team of six trained researchers conducted the interviews in participant’s preferred language (Lusoga, Lugwero, Ateso, Runyankole and Luo). Interviewers and study participants were matched by gender and language. Audio recordings were transcribed verbatim and translated into English. Data analysis followed techniques from thematic and framework analysis [17,18], and also drew upon theories of stigma [19,20], and prior empirical research on barriers to care engagement among PLWH in similar East African settings [21]. In that study, six underlying factors were identified by confirmatory factor analysis to explain barriers to care engagement among out-of-care patients: poverty, inconvenience/work interference, poor treatment/quality at clinic, fear of disclosure of HIV status, healthy family provider/migrant worker, and treatment fatigue/seeking spiritual healing. We used the six-factor framework as a starting point for reduction and synthesis of the data for these analyses. Where new themes were emergent, the team of researchers discussed and modified the existing framework for inclusion of new codes. We drew upon socio-ecologic systems theory [22] for the organization of findings of this analysis.

### Ethical approval

The study received ethical approvals from University of California, San Francisco Committee on Human Research, Makerere University School of Medicine Research and Ethics Committee, Uganda National Council for Science and Technology, and the Ethical Review Committee of the Kenya Medical Research Institute.

## Results

**Fig 1:**
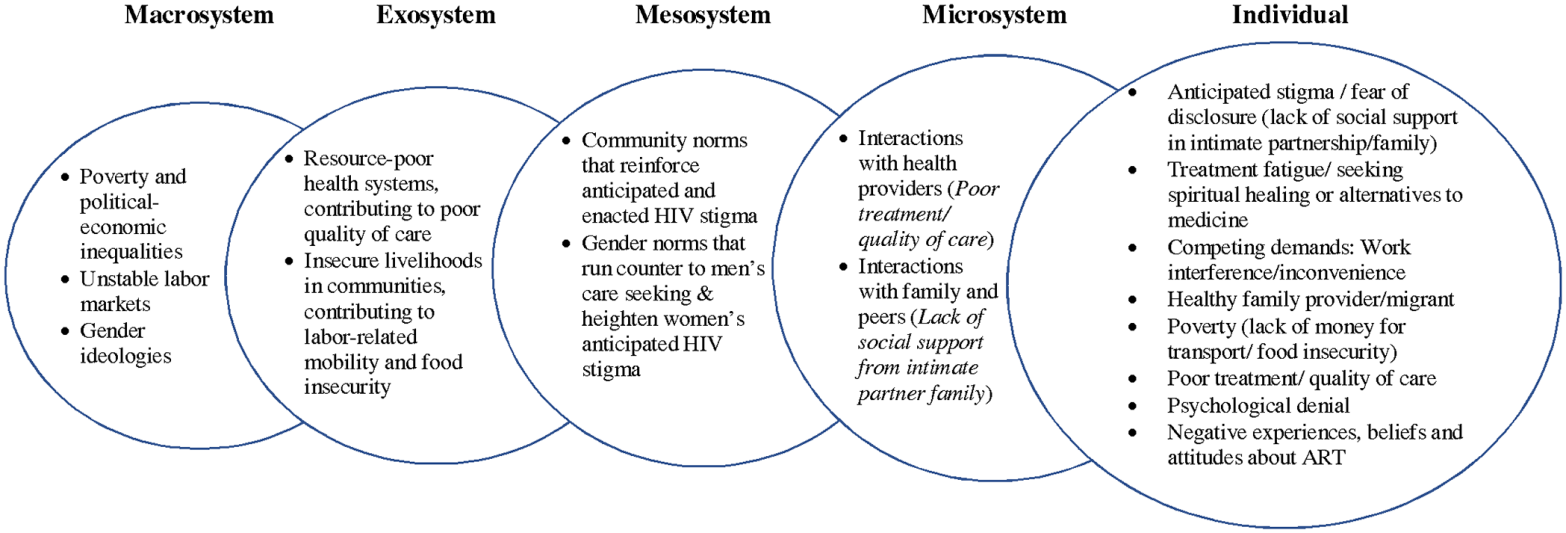
Barriers to care engagement among PLWH organized from distal-to-proximate domains of influence using social-ecological framework. Note: Figure 1 applies emergent findings to distal-to-proximate domains of influence, implied by social-ecological systems theory (Bronfenbrenner, 1979).

In the results below we describe the principal emergent findings related to barriers to engagement in care across the care cascade among the participants. In Fig 1, we have organized these emergent findings into distal-to-proximate domains of influence using a social-ecological framework [22]. The socio-ecological perspective implies that these barriers to care engagement that are experienced by individuals (as documented in the excerpts presented below), are predicated by higher-level domains of influence: the microsystem includes interactions, roles and relations in the individual’s immediate circle of partner, family and peers, including their interactions with health care providers; the mesosystem is made up of interconnections among microsystems, and is often the domain of community norms and values that are perceptible by individuals; the exosystem includes distal systems that affect an individual indirectly, including health systems-level factors that influence quality of care, and socio-economic conditions in settings that affect individuals’ livelihoods, mobility and food insecurity; and finally the macrosystem, which is the broader environmental and political-economic context that influence cultural norms and values, and material conditions. We present direct empirical evidence in support of the individual, microsystem, and mesosystem level influences and barriers to care engagement in the results below. While some barriers appeared to impact upon specific steps in the cascade, others cut across multiple stages (from diagnosis to viral suppression). Gender dimensions to the barriers to care engagement were observed; we describe below how men and women experienced some barriers differently.

### HIV-related stigma

Participants’ narratives revealed that three dimensions of stigma (anticipated, enacted and internalized, or ‘self-stigma’) [19] continue to impede successful engagement in care at every step of the cascade, with its impact felt across the entire HIV care cascade from testing, linkage to, and retention in care due to its diverse manifestations and expressions. PLWH did not want to be seen attending HIV clinics, because of anticipated stigma. Some only felt comfortable accessing care at non-ART clinic times (i.e. after hours) or in settings where no one knew them. Some said they would rather miss their scheduled appointments than meet people who would disclose their status. Clinics were sometimes not set up to help patients to cope with stigma, exacerbating patients’ fears because of inadequate provisions to protect privacy:

“They put it [*the HIV clinic tent*] in a bad location […], in the middle of the compound […] where everyone is watching you go. That tent! Everyone knows that it is for people that have HIV. When I go there, I try to hide, because if someone from this village sees you there, when you come back you find when they have announced to the whole village that you have HIV [*Laughs*]. So it all loses meaning. Whoever brought that virus really killed us.”

*(24*-*year*-*old married female from Uganda*, *engaged in care and on ART)*

Enacted stigma was observed in participants’ accounts of discrimination because of their HIV status. PLWH reported that terms such as “useless”, “dead”, “walking dead”, “finished”, were used to refer to them, and that they were viewed as “promiscuous” and “immoral”:

“They comment and say that people with HIV are moving corpses and are useless.”

*(44*-*year*-*old married male*, *Uganda*, *not engaged in care)*

“They say ‘*so and so has practiced immorality till she has contracted HIV’*. Sometimes I was not involved in immorality but it is my partners who brought in the infection.”

*(27*-*year*-*old divorced female from Kenya*, *engaged in care*, *not on ART)*

“You can hear of some people saying that those who are using ARVs are immoral. They can talk about such in their own groupings, but can also say directly to the infected in case there is a disagreement between them, when one might abuse another while referring to his or her positive status.”

*(46*-*year*-*old divorced female from Kenya*, *engaged in care and on ART)*

Patients perceived that members of their communities viewed HIV infection as a ‘death sentence’ and equated an HIV- positive diagnosis with death:

“They [*community members*] feel that when they have HIV their life has suddenly stopped and death is the only option.”

*(36*-*year*-*old married male from Kenya*, *not engaged in care)*

“Once one has tested positive, then what they think of next is death and this notion is what disturbs the community most.”

*(41*-*year*-*old married female from Kenya*, *engaged in care and on ART)*

PLWH also expressed internalized stigma, as they described feeling judged, ashamed and embarrassed. These feelings sometimes led to depression, low self-worth, and a loss of interest and motivation for self-care.

“People are still feeling embarrassed about the scourge and the majority are still hiding their status.”

*(44*-*year*-*old divorced female*, *Kenya*, *engaged in care and on ART)*

“At times you may feel bad and angry with those people but then you decide to keep quiet. At times you hear a person say in public that you have HIV, and you start to feel embarrassed. You see this disease does not cure and everyone thinks it is the worst disease; in fact the people in my village think that way.”

*(28*-*year*-*old married female from Uganda*, *not engaged in care)*

#### Anticipated stigma: Fear of HIV status disclosure

Fear of disclosure of HIV status is a major manifestation of anticipated stigma, and stood out as a distinct barrier to care engagement, especially at the cascade steps of linkage to and retention in care including drug adherence. Fear of disclosure to spouses or intimate partners as well as other members of the community negatively affected care engagement. These two circumstances are described in turn below:

#### Fear of disclosure to partner

Men feared to be blamed by their spouses for infidelity and bringing “death” to them. Some opted not to engage in care for fear that their spouse may suspect them having HIV if they were to take daily medications, or if they frequently visited the clinic despite appearing well:

“I can say it is my own liking for not having received treatment yet. You know in that house I am living with my wife and the house belongs to the wife, so there is no place I can keep these drugs without her finding out. Exactly, that is what is hindering me [*from enrolling in care*]. I will keep the drugs somewhere and in that house she will definitely get them. Secondly, if I start taking drugs in the morning and evening she will ask me “*I thought you went to the hospital—which disease is this that drugs are just taken continuously*?” So if she asks me like that, how will I respond?”

*(36*-*year*-*old married male from Kenya*, *not engaged in care)*

This barrier appeared to differ by gender, based on commonly held gender role expectations. Men tended to avoid care more than women, sometimes leading to their deterioration and death, while women more often got into care, yet successfully kept their status a secret from their spouses:

“Men do not readily accept HIV care as compared to women. […] Men generally have a difficult understanding on life matters as compared to a woman…”

*(38*-*year*-*old widowed female*, *Kenya*, *engaged in care and on ART)*

#### Fear of disclosure at clinics

Out of fear to be labeled with derogatory tags such as “corpses” or “dead”, individuals with HIV choose to only disclose to a few individuals whom they perceived as safe custodians of their “secret”. This presented a challenge in seeking care and getting drug refills, as individuals did not want other members of the community to know their status—especially in the early stages of accessing care in HIV clinics:

“… At first I was scared of going there [*the local HIV clinic*] to meet people there that I know or that know me, but I did not have transport go to Mbarara—I would not be saving any money. I decided that come what may, because I am not the only one that has HIV [*I would seek treatment at the local clinic*]. However, when I board a motorbike, I tell the person riding me to take me to town—I do not tell him to take me directly to the health center. So when I get to town, I walk to the health center on foot so that no one sees me.”

*(41*-*year*-*old married female from Uganda*, *engaged in care and on ART)*

### Denial

Denial of HIV-positive status after testing was a major barrier to linkage to and retention in care and resulted in delayed initiation of treatment, with some refusing to take their medication after being initiated on ART. Some individuals described having been in psychological denial, while others described having avoided care more as a manifestation of negative coping.

“Some normally wait to the final stages of HIV/AIDS for them to act and they must be pushed hard to do so.”

*(31*-*year*-*old widowed female from Kenya*, *engaged in care and on ART)*

“[*I took*] about five years [*before enrolling into care*] and in between I used to go for repeated tests which all tested positive.”

*(42*-*year*-*old married male from Kenya*, *engaged in care and on ART)*

“…Because I was in denial, I threw the drugs in the water while I was on my way home from the hospital. I later became sicker and my mother-in-law now talked to the doctors to come for me since she did not want to lose yet another person in the family after the son [*my husband*] had passed on.”

(*41 year*-*old widowed female*, *Kenya*, *engaged in care and on ART*)

Participants said that men, especially young men, were disproportionately affected by denial:

“Men will only visit the hospitals when critically sick […] Most of them [those who die as a result of HIV] are young men”

*(38*-*year*-*old widowed female*, *Kenya*, *engaged in care and on ART)*

### Poverty

Poverty strongly affects linkage to and retention in care, both through food insecurity and also difficulty with paying for transportation to and from clinic. PLWH require adequate nutritious food for best outcomes on treatment, and patients report aggravated side effects when medications are taken on an empty stomach. Poverty, as a driver of food insecurity, therefore emerged as a salient barrier to drug adherence:

“I don’t like taking these drugs when there is no food. When I have nothing to eat, I don’t take them…”

*(36*-*year*-*old married female*, *Kenya*, *engaged in care and on ART)*

Secondly, HIV is a chronic condition and requires money for transportation for multiple follow-up visits at health facilities:

“Transport is a burden. At times you do not have money and you have exceeded your appointment dates. […] It is only transport that disturbs me, because I do not have where to get it from.”

*(24*-*year*-*old married female*, *Uganda*, *engaged in care and on ART)*

### Poor quality of health services

A hostile treatment environment, including negative attitudes from healthcare providers and long wait times, was also found to hinder retention in care. Some participants described encounters with health care providers with a ‘bad attitude’, who humiliated patients. This resulted in some patients stopping care, and others changing health facilities:

“These service providers are sometimes not friendly […] The providers make noise to the clients who have either come late or defaulted and send them back home without drugs. This sometimes is humiliating to the clients who sometimes get demoralized and just stop going for drugs completely.”

*(31*-*year*-*old widowed female*, *Kenya*, *engaged in care and on ART)*

“I know that they [*antiretroviral medications*] are really helping people a lot. I also know that they are harmful if not used correctly, though this could come as a result of many factors, like distance to the health facility and even the way the providers handle those who have defaulted. They are punished by the facility for defaulting. They either go without drugs, or quarreled [*with*] by the providers, and this in turn discourages people—they just continue defaulting because they do not want to go through the negative attitude from the providers.”

*(41*-*year*-*old divorced female from Kenya*, *engaged in care and on ART)*

Some viewed the long clinic visit due to wait times as an additional expense, since they have to spend additional money on a meal while waiting to be attended to:

“We spend a lot of time at the health facility. If you do not have money to buy tea and food, you can collapse.”

*(28*-*year*-*old single male*, *Uganda*, *engaged in care and on ART)*

### Competing demands: Care-seeking interfering with work

Engagement in chronic care is demanding in this high-poverty, rural setting, both in terms of time and money spent in attending clinic appointments. Attending clinic may take a whole day and thus interfere with income generating activities; patients had to weigh losing a day’s income against attending a clinic appointment. In addition, the livelihoods that patients are involved in, such as fishing, sometimes involved expeditions that took days or weeks, presenting a challenge to drug adherence:

“… sometimes I may leave for the [town] center with the aim of not going to the lake knowing that[…]I will go back home and take my drugs—but I find people at the center who force me to take them inside the lake. Something I didn’t prepare for….”

*(38*-*year*-*old married male*, *Kenya*, *engaged in care and on ART)*

“The fact that I must collect my drugs sometimes bar me from undertaking some personal activities, but I must collect them because this is where my help comes from.”

*(30*-*year*-*old married male from Kenya*, *engaged in care and on ART)*

### Feeling healthy

Feeling healthy, especially among those whose health had improved after ART, also emerged as a barrier to retention in care and drug adherence. Some dropped out of care because they felt that they did not need the medication any longer:

“… there is somebody who truly is sick and has been on drugs for two years—but because he thinks that he has recovered, since he is looking healthy and feeling normal […], [*he*] puts drugs aside. Taking the dose morning and evening is not easy.”

*(45*-*year*-*old married male*, *Kenya*, *engaged in care and on ART)*

“These people just refuse going to the hospital for drugs because they think that they are now healthy. Others just stop because they are tired of taking drugs”

*(31*-*year*-*old married female from Kenya*, *engaged in care and on ART)*

### Treatment fatigue

For a lifelong condition requiring daily oral medication, treatment fatigue is a key barrier. Some PLWH referred to themselves as “prisoners” to the medication. This theme emerged as a barrier to drug adherence, a necessary component for viral suppression. Some got tired along the way and stopped their medication altogether:

“It is like being in a jail, because it has to be taken daily.”

*(49*-*year*-*old married male*, *Kenya*, *engaged in care and on ART)*

“We feel like prisoners to the drugs, because all the time we are supposed to have them with us no matter where we are and where we go, as well as to adhering to the time, which is the greatest challenge.”

*(45*-*year*-*old married female from Kenya*, *engaged in care and on ART)*

### Drug side effects

Participants reported experiencing side effects especially at the beginning of treatment, which subsided in most cases. The severe or protracted forms are most worrying to patients, and without reassurance, patients were likely to stop their medications hampering drug adherence, with resultant viral non-suppression:

“….When I swallowed these drugs for the first time, I used to have bad dreams and get nausea[…] I used to feel so weak and I would collapse and so I called the providers.[…] I told them […] ‘it seems these drugs that you gave me are going to kill me.’”

*(30*-*year-old married male*, *Uganda*, *engaged in care and on ART)*

### Negative beliefs and attitudes about ART

Firmly held beliefs, whether true or false, and negative experiences emerged as barriers to linkage to care, ART initiation and drug adherence. Most of the beliefs were centered around ART, mostly associated with negative effects to the patient on treatment.

“I was in a group of 30 members who all died in succession only three of us are still alive. This made me plead with the doctors not to initiate me on ARVs since I knew those on ARVs die sudden deaths.”

*(51*-*year*-*old married male*, *Kenya*, *engaged in care and on ART)*

## Discussion

This qualitative study documented a conceptually-coherent set of factors hindering positive outcomes at three stages in the HIV care cascade proposed to yield greatest impact in shutting down the HIV epidemic, per the UNAIDS 90-90-90 targets. These ranged from psychosocial factors such as dimensions of HIV-related stigma, especially as manifested in fear of disclosure that hindered individuals from being seen at HIV clinics as has been documented previously [23,24], to structural factors related to poverty such as food insecurity and a lack of money for transport, to health systems barriers which hindered drug adherence and clinic retention. In some instances these factors were inter-related, as viewing clinic attendance as a competing demand that interfered with income-generating activities interacted with health systems factors such as long wait times (a marker of poor quality of care).

These qualitative findings mapped well to a previously empirically-derived set of factors explaining barriers to care engagement among out-of-care patients, using health systems data from programs in several East African countries [21] and provide theoretical validation of this framework to explain barriers to care engagement. The empirically-derived six-factor structure was not, however, comprehensive; this qualitative study suggested that in addition to the key six domains previously identified (anticipated stigma and fear of disclosure; treatment fatigue, and seeking spiritual healing or alternatives to medicine; competing demands, or work interfering with care-seeking; being a healthy provider for one’s family, often as a migrant; poverty, manifested as lack of money for transport and food insecurity; and poor quality of care at clinics), there were two additional emergent themes related to barriers: psychological denial/negative coping; and having negative experiences (including side effects), beliefs and attitudes about ART. Further work should be done to develop instruments to predict threats to care engagement that can be used by health care workers to identify patients at risk of dropout, or by health systems to evaluate progress towards addressing barriers to care engagement in resource-poor settings.

While both men and women in the communities experienced high levels of anticipated stigma, our study reveals a gender dimension to these barriers. We found that, while both men and women feared disclosure, women more readily engaged in care despite the fact that the negative consequences of disclosure that they anticipated (abandonment or violence) were more severe. Men avoided care more readily than women in response to fears of being blamed and shamed. In addition, men’s livelihoods that involved mobility (e.g. fishing), combined with male gender norms that reinforced men’s needs to uphold their status as breadwinners, made perceived work interference a particularly potent barrier. These findings are consistent with those we have previously described in a larger qualitative study of HIV status disclosure experiences in SEARCH communities [25].

Our findings underscore the persistence of HIV-related stigma in rural East African settings. Though rapid advancements have been made in HIV treatment, negative perceptions associated with HIV disease have not evolved as rapidly [26,27]. Our study confirms findings of other recent studies [23,24] that stigma remains a major barrier to successful care engagement. Various approaches are needed to directly address stigma, including stronger social support structures for patients, clinic integration, spaced out clinic appointments for stable patients, and mental health care services that have been shown to improve retention [28,29]. Other interventions, including education programs to enhance understanding of HIV disease, connecting PLWH with their peers and the community, and skills-building through peer coaching, have shown some promise for addressing stigma [30,31,32].

Quality of care, including patient-provider interactions, is an important facilitator of care engagement that is amenable to intervention. Our findings were consistent with prior studies showing that poor health services, health provider attitudes and clinic set-ups deterred care engagement [23,24]. Patient-friendly services are needed, accommodating patient desires such as confidentiality, flexibility in clinic appointments and friendly interactions with patients [23]. Streamlined care models designed to provide more flexible, patient-centered care have been shown to produce favorable outcomes among HIV patients in diverse settings [33].

Other factors may pose a more stubborn threat to successful care engagement, including treatment fatigue. Therapy simplification including alternative drug formulation as well as reduced frequency of drug dosing and pill burden are known to improve adherence to treatment translating to better outcomes in HIV management [34]. First line treatment with one pill a day and development of injectable long acting ART are hopeful developments in this respect, more still needs to be done to address this concern.

Our study was subject to limitations. This analysis drew information from PLWH, and did not include perspectives from other parties such as healthcare providers, family members or other community members. However, we view this focus as a necessary step to ensure conceptual rigor in analyzing themes emergent from the data, foregrounding voices and experiences of PLWH. Interviews are susceptible to social desirability bias. Our interviewers were trained to reaffirm confidentiality and avoid judgmental reactions, which aided in minimizing this bias. Our study does not present data on certain key populations such as men who have sex with men (MSM), sex workers and injection drug users, data that may be of interest in other settings due to unique care engagement barriers experienced by these groups. The scope of this analysis was limited to barriers of care engagement, and did not include analyses of facilitators, permitting us to present findings in depth. The findings are strengthened by the breadth and regional heterogeneity of our data sources, composed of interviews with PLWH living in communities across three regions of Uganda and Kenya; thus, findings are potentially applicable to other rural settings in developing countries. With respect to the reflexive nature of this research, the investigators in this study comprised a multidisciplinary cross-regional team including African researchers living the study settings, an asset for inclusion of multiple perspectives in the interpretive process. Finally, this analysis explores barriers along the entire cascade of care, demonstrating the impact of specific barriers across several points in the care cascade, as well as their distal to proximate levels of influence.

## Conclusions

Achieving the 90-90-90 targets promises both to optimize individual health and prevent onward HIV transmission. As universal treatment is expanded in sub-Saharan Africa it will be essential to address the barriers at critical steps of the HIV care cascade. Sustained efforts to decrease HIV-related stigma as a major impediment to meaningful engagement are still required. Additionally, health care systems and policy makers must make deliberate efforts to deliver patient-centered, patient-sensitive and patient-responsive care to promote sustained engagement.
